# Assessing the Role of Gaseous Chlorine Dioxide in Modulating the Postharvest Ripening of Keitt Mangoes through the Induction of Ethylene Biosynthesis

**DOI:** 10.3390/foods13020316

**Published:** 2024-01-19

**Authors:** Dongwei Zhang, Binxiong Liu, Shaoyi Wu, Changcheng Li, Ting Fang, Meiling Tian

**Affiliations:** 1 College of Food Science, Fujian Agriculture and Forestry University, Fuzhou 350002, China; zhangdongwei0321@163.com (D.Z.); lbx_zhou@163.com (B.L.); wsy690049406@gmail.com (S.W.); changcheng_li@fafu.edu.cn (C.L.); 2 National R&D Center for Vegetable Processing, Fuzhou 350002, China

**Keywords:** Keitt mango, gaseous chlorine dioxide, fruit ripening, ethylene biosynthesis

## Abstract

Consumer acceptance of Keitt mangoes (*Mangifera indica* L.) is significantly affected by their slow postharvest ripening. This work used gaseous chlorine dioxide (ClO_2_(g)) to prepare the ready-to-eat Keitt mango and explored the potential mechanisms for the mango ripening. Harvested mangoes were treated with 20 mg·L^−1^ of ClO_2_(g) or ethephon for 3 h (25 °C) and left in a climatic chamber with a temperature of 25 ± 1 °C and a relative humidity of 85 ± 5% for 4 d. The results showed that ClO_2_(g) treatment significantly promoted the orange coloration of mango flesh compared to the untreated control group. Moreover, ClO_2_(g) treatment significantly elevated the total soluble solids, total soluble sugar, and total carotenoids content of mangoes, whereas the firmness and titratable acidity were reduced. ClO_2_(g)-treated mangoes reached the edible window on day 2, as did mangoes treated with ethephon at the same concentration, except that the sweetness was prominent. The residual ClO_2_ level of the mangoes was <0.3 mg/kg during the whole storage time, which is a safe level for fruit. In addition, ClO_2_(g) significantly advanced the onset of ethylene peaks by 0.5 days and increased its production between days 0.5 and 2 compared to the control group. Consistently, the genes involved in ethylene biosynthesis including *miACS6*, *miACO1*, and *miACO* were upregulated. In sum, ClO_2_(g) can be a potential technique to reduce the time for harvested mango to reach the edible window, and it functions in modulating postharvest ripening by inducing ethylene biosynthesis.

## 1. Introduction

Mango (*Mangifera indica* L.) is the second most widely cultivated fruit crop in many tropical and subtropical countries [1]. Mango is globally admired due to its pleasant aroma, juicy nature, and high nutrition content [2,3,4]. Keitt mango is a late-ripening mango cultivar native to Florida that matures in August and September [5]. In late November, the mangoes are still available on the market when the supply of mangoes from Myanmar, Vietnam, India, Guangxi, and Hainan comes to an end, which compensates for the increased demand for mangoes in the market during this period [6]. In China, the unique location of Panzhihua in Sichuan provides good conditions for producing Keitt mango [6]. The cultivation area of Keitt mango in Panzhihua has exceeded 40,000 hectares, with a production of 220,000 tons in 2020 [7]. Nevertheless, Keitt mango is a climacteric fruit characterized by postharvest ripening. Therefore, it is commonly harvested at full-sized mature green (unripe and inedible) to ensure its quality, transport, and storage [8]. Consumers must wait 4–7 days for the edible Keitt mango. With the expansion of the fresh food market and the changes in consumption habits, ready-to-eat fruit has become a new market demand in China [9]. 

To prepare the ready-to-eat Keitt mango and improve its consumption appeal, mangoes are currently commercially ripened using artificial ripening agents such as ethylene and ethylene-generating reagents (ethephon) under controlled conditions [10]. Ethylene is the first identified gaseous plant hormone and its role in fruit ripening has been fully demonstrated [11,12,13]. Classically, ethylene is perceived by ethylene receptors (ETRs), leading to the inactivation of constitutive triple-response1 (CTR1) and the activation of ethylene insensitive2 (EIN2) that stabilizes EIN3/ethylene insensitive3-like (EIN3/EIL) transcription factors, resulting in the expression of ethylene-responsive genes such as ethylene-responsive factors (ERFs), which ultimately promote fruit ripening [14]. Ethephon is an effective substitute for ethylene gas in production. It acts to penetrate the fruit and break down into ethylene, which regulates the ripening of the mango [15]. ABA has been reported to be a critical ripening agent for non-climacteric fruits [16,17]. Recently, research demonstrated that ABA can promote climatic fruit (mango) ripening by acting as a stimulator to trigger ethylene biosynthesis or by directly regulating the basic leucine zipper (bZIP) transcription factor (TF) MiHY5 [18,19]. Nevertheless, fruits ripened with ethylene have been associated with poor flavor development and tend to spoil quickly [20]. Ethephon and ABA were commonly administered in the form of liquid immersion [19], and the high-humidity environment can predispose fruits to microbial spoilage [21]. These factors severely affect the commodity value of Keitt mango and reduce the likelihood of consumers repurchasing. Therefore, new ripening techniques are urgently needed to ripen the Keitt mango and maintain its quality. 

Gaseous chlorine dioxide (ClO₂(g)) is categorized as a Class A1 safe and efficient disinfectant by the WHO, with strong oxidation and antibacterial activity [22]. The use of ClO₂(g) for postharvest preservation has been of increasing interest in recent years due to its advantages such as powerful antimicrobial activity, rapid diffusion, less corrosive, low environmental pollution, and fewer residues on edible fractions of fruits and vegetables [22]. ClO₂(g) (3.5–22 mg·L^−1^) can reduce the populations of spoilage organisms (such as *Escherichia coli* and *Alternaria alternata*), inhibit the ethylene release, and decrease the ripening-related enzyme activity of tomato fruit, thus achieving a certain preservation effect [23,24]. Consistently, our previous study developed a novel ClO₂(g) treatment system and demonstrated a significant protection and preservation effect on the safety and quality of table grapes in cold storage [25]. Nevertheless, high concentrations of ClO₂(g) may promote fruit ripening due to its high oxidative properties [26]. A 30 mg·L^−1^ ClO₂(g) treatment significantly increased the content of reducing sugars in grapes [26]. In our preliminary experiment, we found that ClO₂(g) promotes the softening of Keitt mango fruits, which is an important indicator of fruit ripening. Moreover, the use of ClO₂(g) in mangoes allows for simultaneous sterilization and ripening, thus providing a certain safety for ready-to-eat mangoes. However, the effect of ClO₂(g) treatment on the quality attributes associated with postharvest ripening of Keitt mango and the underlying mechanism has not been systematically studied. 

In the current study, we investigated the impact of ClO₂(g) treatment on the physical and biochemical quality associated with the ripening of Keitt mango fruit under room temperature (25 °C) storage conditions. Moreover, the potential mechanism of ClO₂(g) treatment on Keitt mango ripening was explored using transcriptomics and quantitative real-time PCR techniques. This investigation provides a technical basis for improving the quality of ready-to-eat Keitt mango fruit and elevating its commercial value.

## 2. Materials and Methods

### 2.1. Mango Fruit

Mature green stage Keitt mangoes were obtained from a local orchard in Panzhihua City, Sichuan Province, China. The mango fruits were hand-picked, with field heat dissipation, packed in Styrofoam boxes, and transported to the postharvest laboratory via cold chain logistics on the same day. The average weight of the fruits used in this study was 1050 ± 150 g. The peel of the fruit should be free from any damage and visible symptoms of disease and mechanical injury. 

### 2.2. Procedure and Experimental Design

#### 2.2.1. Gas ClO_2_ Preparation

**Figure 1:**
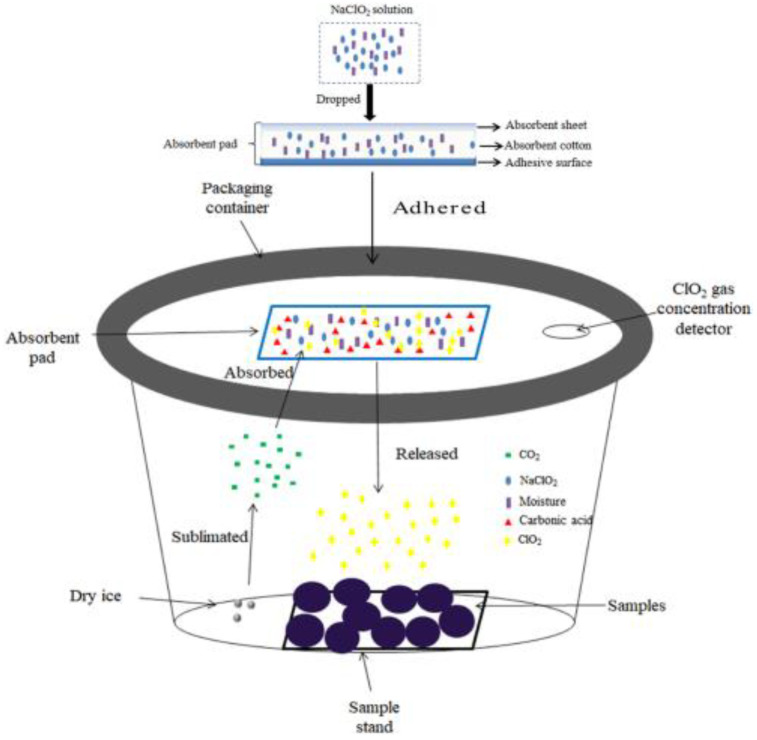
Schematic of gaseous ClO_2_ treatment system [25].

The gaseous ClO₂ was obtained using a novel gaseous chlorine dioxide treatment system developed by our previous study, and the schematic of the ClO_2_(g) treatment system is shown in Figure 1 [25]. Briefly, 0.4 mL of 20% NaClO_2_ solution was injected in equal amounts into four absorbent pads (1 cm × 1 cm × 1 cm; CoSoFth Co., Jinjiang, China), which were affixed to the inside wall of a self-made treatment chamber (15 L). Then, CO_2_ gas was generated using 5 ± 0.2 g of dry ice (BaiQuan Co., Fuzhou, China). The CO_2_ gas was absorbed by the absorbent pads and dissolved in the solution to form H_2_CO_3_, which then reacted with NaClO_2_ to produce ClO₂ gas. The equations for ClO₂ gas generation are as follows. The intensity of chlorine dioxide gas is calculated by the cumulative exposure of chlorine dioxide (mg·L^−1^).

(1) $${CO}_{2}(g)+H_{2}O↔H_{2}{CO}_{3}$$

(2) $$2H_{2}{CO}_{3}+5{NaClO}_{2}\to 2H_{2}O+2{Na}_{2}{CO}_{3}+4{ClO}_{2}(g)+NaCl$$

#### 2.2.2. Design of the Experiment

Mango fruits were randomly divided into three groups, each with with 50 biological replicates. Mangoes in the ClO_2_(g) group were placed on the sample stand in the chamber and exposed to the ClO_2_ gas (20 mg·L^−1^) for 3 h. In the control group, mangoes were exposed to the air for 3 h in the same chamber. In the ethephon group (used as the positive control), mangoes were sprayed with 1 mL of ethephon (20 mg·L^−1^) and incubated for 3 h in the chamber. Three sets of mangoes were then placed in separate artificial climate chambers (RXZ-280B, Jiangnan Ltd., Ningbo, China) and left to ripen at 25 ± 1 °C and 85 ± 5% relative humidity for four days. Ten mangoes were sampled at a time on days 0.5, 1, 2, 3, and 4. Moreover, 10 mangoes without any treatment were randomly sampled to assess their initial fruit quality. The whole experiment was repeated three times.

#### 2.2.3. Observed Response

The color change of the peel of each sampled mango was observed. After cutting the mango longitudinally, the mango flesh color change was observed and photographed.

### 2.3. Fresh Weight Loss

Fresh weight loss was evaluated at every sampling interval and calculated according to the following formula.

(3) $$Fresh\;weight\;loss\left(\%\right)=\frac{Initial\;weight-Final\;weight}{Initial\;weight}\times 100$$

### 2.4. Quality Attributes

Six mangoes from each group were taken at a time on days 0.5, 1, 2, 3, and 4, and the following quality attributes were measured and repeated three times.

#### 2.4.1. Sensory Analysis

To evaluate the sensory profiles of mango samples, fifteen panelists with experience in mango consumption were trained according to the method described by Silué et al. [27]. An ethical approval (code PZCASFAFU 23043, approval date 9 March 2023) was given for this sensory research by the Academic Committee of Fujian Agriculture and Forestry University. Descriptors used for the evaluation included overall rating, aroma, pulp color, chewiness, sweetness, and sourness. The samples were served on disposable plates and coded by two numbers. Panelists were asked to rate descriptors on a scale of 0–15, with 0 indicating “no perception” and 15 indicating “extremely intense”. Between assessments, the panelists’ mouth was washed with spring water and wiped with a paper towel. The evaluation was carried out in a room with daylight. 

#### 2.4.2. Color Measurement

The peel and pulp color of mango samples was assessed using a colorimeter (Model CR-20, Konica Minolta, Tokyo, Japan) and presented in the values of lightness (L*), green chromaticity (a*), yellow chromaticity (b*), and hue angle (h*). The color of the peel and pulp was measured in the middle of the surface and the inner side of the cheek after cutting the mango lengthwise along the core.

#### 2.4.3. Firmness

Firmness was measured by a textural analyzer (BaoSheng Co., Shanghai, China) with a 5 mm diameter probe penetrating 5 mm through the top central portion of the sample at a speed of 1 mm·s^−1^. The average firmness value was calculated using values taken at six points over an equal area of each fruit. The six points were selected on the internal tissue of the isometric axis of the peeled mango, four at the ends and another pair in the center. Firmness was expressed as Newton (N). 

#### 2.4.4. Titratable Acidity, Total Soluble Solids, and Total Starch Content

The titratable acidity (TA) of mango pulp was determined by titrating 20 mL of mango pulp juice with 0.1 mol/L NaOH until neutralization of the organic acids [2], and the results were expressed as citric acid content in grams per kilogram. The total soluble solids (TSS) were measured using a refractometer (Model HP-TD1, AnYing Co., Yantai, China) and expressed as %Brix. Total starch content was determined using the total starch kit (K-TSTA, Megazyme, Bray, Ireland) according to the manual instructions and expressed as % starch relative to the weight of the sample.

#### 2.4.5. Total Soluble Sugar

The total soluble sugar content of mango pulp was determined using the anthrone sulfate method [28]. Briefly, the total soluble sugar of mango pulp was extracted using boiled deionized water and centrifuged at 8000× *g* for 10 min to obtain the supernatant. Then, the supernatant was mixed with ethyl acetate and H_2_SO_4_, boiled for 1 min, and measured at 630 nm using a Microplate Reader (Thermo Scientific, Multiskan SkyHigh, Marsiling Rise, Singapore). The concentration of the total soluble sugar was calculated based on a standard curve of sucrose (Sigma-Aldrich, St. Louis, MO, USA)

#### 2.4.6. Total Carotenoids

The total carotenoid content of mango pulp was determined by the method proposed by González-Casado et al. [29] with slight modifications. The milled mango pulp was homogenized with 80% acetone (*v*/*v*), incubated at 25 ± 2 °C for 24 h protected from light, and centrifuged at 12,000× *g* for 10 min at 4 °C. The supernatant was measured at 470 nm, and the total carotenoid content was calculated as follows.

(4) $$Total\;carotenoid\;content(mg\cdot {kg}^{-1})=\frac{A_{470}\cdot V\cdot 10^{4}}{A_{1cm}^{1\%}\cdot M}$$

where A_470_ is the absorbance of the sample at 470 nm; V is total extract volume (mL); $A_{1cm}^{1\%}$ is the extinction coefficient of carotenoid mixtures, 2592; and M is the sample weight (g). The total carotenoid content in mango pulp was expressed as mg/kg.

### 2.5. Residual ClO_2_ Analyses

The mango fruit or pulp sample was immersed with 1000 mL of deionized water in the sterile sealing bag and shaken for 5 min. An amount of 10 mL of solution was taken from each sterile sealed bag, and the ClO_2_ residue was analyzed using a Q/HK 080318 ClO_2_ colorimeter (HuaiKai Co., Guangzhou, China) with the DPD (N, N-diethyl-p-phenylenediamine) method [30]. The residual ClO_2_ level was expressed as mg/kg. The measurement was repeated three times, and each replicate contained 6 fruits.

### 2.6. Ethylene Production, Respiration Rate, and Abscisic Acid Content

The ethylene content was determined using a sensor sense laser ethylene detector (ETD-300, Nijmegen, The Netherlands) with a gas handling system [31]. One fruit from each replicate was placed in a closed bag and continuously flushed with air at a flow rate of 4 L/h. Each sample was monitored in real-time for 15 min. The ethylene release rate was calculated as the average of the last 10 min of ethylene production in nmol C_2_H_4_ kg^−1^ s^−1^. The respiration rate was measured by titrating the CO_2_ produced by mangoes and absorbed in the 0.4 mol/L NaOH with 0.3 mol/L H_2_C_2_O_4_ [32]. The respiration rate was expressed as mg CO_2_ kg^−1^ h^−1^. The content of abscisic acid in mango pulp was determined using an ELISA kit (ZC-53364, Zci Bio, Shanghai, China) according to the manufacturer’s instructions and expressed as mg ABA g^−1^. The contents of ethylene and ABA were recorded for each group on days 0.5, 1, 2, 3, and 4, with three biological replicates at each time point.

### 2.7. RNA Sequencing (RNA-Seq) Analysis

RNA-seq analysis was performed by Majorbio Bio-pharm Biotechnology Co., Ltd. (Shanghai, China). In brief, the total RNA of mango pulp was extracted using Plant RNA Purification Reagent (Invitrogen, Waltham, MA, USA) according to the manufacturer’s instructions, and genomic DNA was removed using DNase I (Perfect Real Time; TaKaRa, Tokyo, Japan). Subsequently, RNA quality was determined by 1% agarose gels, 2100 Bioanalyzer (Agilent Technologies, Wilmington, DE, USA), and NanoDrop-2000 (Thermo Fisher Scientific, Wilmington, DE, America). RNA samples that meet the requirements for library construction were purified, reverse transcribed, library constructed, and sequenced. The genes with the parameter of false discovery rate (FDR) ≤ 0.05 and absolute fold change ≥ 2 were considered differentially expressed genes (DEGs). Transcript abundance was quantified using Transcripts Per Million reads (TPM). The principal component analysis (PCA) algorithm was conducted using the scikit-learn library of the Python software package (3.11 version). GO functional enrichment and KEGG pathway analysis were conducted using Goatools (https://github.com/tanghaibao/Goatools. Accessed on 11 January 2024) and KOBAS (http://kobas.cbi.pku.edu.cn/home.do. Accessed on 11 January 2024). All raw sequencing data have been deposited in the Gene Expression Omnibus (GEO) of NCBI with the accession number PRJNA985857 (data will be released on 1 January 2025). Four biological replicates were used for RNA-seq analysis in each group.

### 2.8. Quantitative Real-Time PCR (RT-qPCR) Analysis 

Total RNA from the mango pulp was extracted as described in Section 2.7 of this study and further transcribed into complementary DNA (cDNA) using transcriptor reverse transcriptase (TakaRa Bio, Inc., Shiga, Japan). Primer sequences were designed using NCBI/Primer-BLAST (https://www.ncbi.nlm.nih.gov/tools/primer-blast/. Accessed on 11 January 2024), and they are listed in the Supplementary Table S1. The RT-qPCR analysis was performed on a C1000^TM^ Thermal Cycler PCR detection system (Bio-Rad, CFX96^TM^ Optics Module, Morgan Hill, CA, USA) using protocols described previously [16]. The expression levels of the target genes were normalized to the expression of β-actin and quantified using the 2^−ΔΔCt^ method. Three replicates were used for the quantification with three biological replicates at each group. 

### 2.9. Statistical Analysis

Data were presented as the mean ± SD. Unless otherwise stated, all measurements for each analysis were performed in triplicate, and each analysis contained 5 fruits. The differences between groups were analyzed by SPSS software (27.0 version, SPSS Inc., Chicago, IL, USA) using one-way ANOVA post hoc Duncan multiple range tests and considered significant at *p* < 0.05. Figures were plotted using GraphPad Prism software (version 9.0, San Diego, CA, USA).

## 3. Results

### 3.1. Physical Quality Characteristics of Mango Fruits

**Figure 2:**
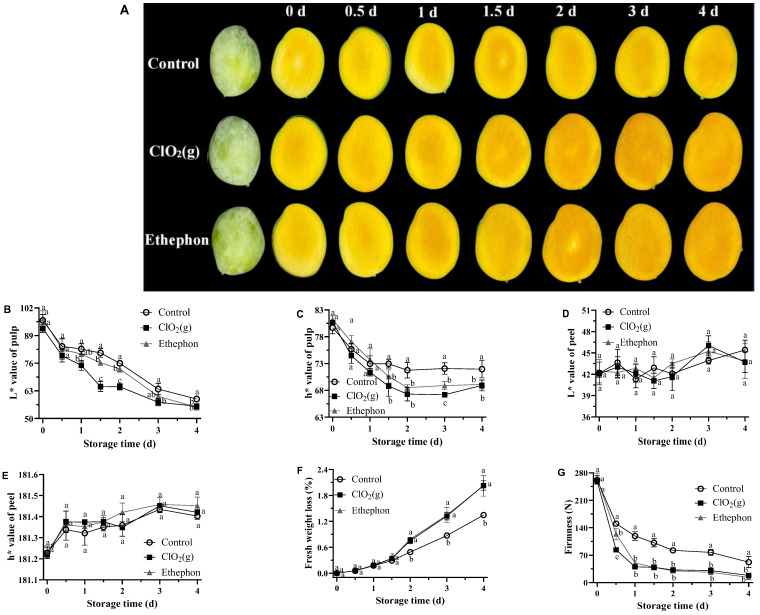
Physical quality characteristics of untreated, ClO_2_(g)-treated, and ethephon-treated mangoes during storage at 25 °C. Visual appearance changes (**A**); L* value (**B**) and h* value (**C**) of mango pulp; L* value (**D**) and h* value (**E**) of mango peel; fresh weight loss of mango fruit (**F**); firmness of mango pulp (**G**). Different letters represent statistical differences between groups (*p* < 0.05).

The pulp color changes in the mangoes treated with air (control group), ClO₂(g), and ethephon during the storage time are shown in Figure 2A. All three groups of mangoes turned orange with the storage time. The ClO₂(g) treatment was visually as effective as ethephon in promoting the orange coloration of mango flesh and the color was darker than that of the control group from day 2 to day 4 (Figure 2A). The L* and h* values progressively decreased with an advanced storage period in all groups. The decreased L* and h* values suggested that the color of mango pulp became darker and developed a yellow-orange color. ClO₂(g) and ethephon treatment significantly reduced the L* and h* values from day 2 to day 4 (Figure 2B,C). In contrast, ClO₂(g) treatment caused no significant peel color change in the mango skin throughout the storage period (Figure 2D,E). The color changes of mango pulp and skin indicated that ClO₂(g) treatment accelerated mango postharvest ripening without changing its appearance.

Fresh weight loss was observed in both treated and untreated mango fruits during postharvest ripening at 25 °C (Figure 2F). The weight loss of mango fruits treated with ClO₂(g) and ethephon increased with the storage days and reached a maximum of 2.03% and 2.02%, respectively, on day 4 (Figure 2F). A weight loss between 7 and 9% is the maximum acceptable weight loss for mangoes before they become unsellable [33]. Our results suggested that the weight loss caused by ClO₂(g) treatment was acceptable. 

Fruit firmness is a major indicator for evaluating mango maturity [34]. Keitt mangoes reached an edible window with firmness of 25–35 N [35]. In our study, the firmness of mango pulp was continuously decreased in all treatments throughout the storage period (Figure 2G). The firmness of ClO₂(g) and ethephon-treated mango pulp was significantly lower than that of the control group after 12 h of storage (Figure 2G). On day 2, the firmness of mango pulp treated with ClO₂(g) and ethephon was 32.43 N and 30.28 N, respectively, while the firmness in the control group was 82.43 N (Figure 2G). These results suggest that ClO₂(g) application significantly reduces the time for Keitt mangoes to reach the edible window.

### 3.2. Biochemical Quality Attributes of Mango Fruits

**Figure 3:**
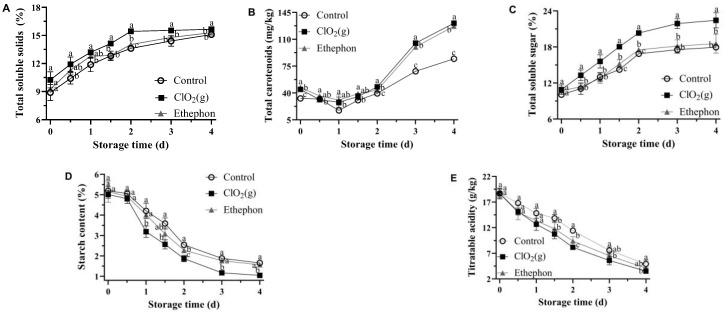
Biochemical quality attributes of untreated, ClO_2_(g)-treated, and ethephon-treated mangoes during storage at 25 °C. The contents of total soluble solids (**A**), total carotenoids (**B**), total soluble sugar (**C**), starch (**D**), and titratable acidity (**E**) in mango pulp. Different letters represent statistical differences between groups (*p* < 0.05).

The TSS, total soluble sugar, and total carotenoids of mango pulp increased progressively in all treatments throughout the storage period, whereas starch and TA decreased with storage time (Figure 3). These changes were consistent with the characteristics of ripening mangoes [36]. The application of ClO_2_(g) significantly promoted the increase of TSS, total soluble sugar, and total carotenoid content of mango pulp from day 1 (Figure 3A–C). The Keitt mango reached an edible window with a TSS of 14–18% [35,37]. The TSS of mangoes treated with ClO₂(g) reached 14% on day 1.5, those with ethephon treatment on day 2, and the control group on day 3 (Figure 3A), which further suggested that ClO₂(g) treatment can reduce the time of Keitt mangoes to reach the edible window.

Mango treated with ClO₂(g) and ethephon showed a substantially higher accumulation of total carotenoids from day 2 to day 4 as compared to the control group (Figure 3B), which was consistent with the previous findings that carotenoids accumulated exponentially during mango ripening [38]. The total carotenoid content of mango pulp treated with ClO₂ (g) and ethephon was 1.98-fold and 1.96-fold higher on day 4, respectively (Figure 3B). In addition, a significant difference in the total soluble sugar content between ClO₂(g) and the other treatments was observed (Figure 3C). On days 2, 3, and 4, the total soluble sugar content in mango pulp treated with ClO₂(g) was noticeably higher (1.2-fold) than the other treatments (Figure 3C). 

On the other hand, on day 2, ClO₂ (g) treated mango pulp showed 18.12% and 26.54% reduction in starch content compared to ethephon and control groups, respectively (Figure 3D). The reduction of starch during fruit ripening leads to an increase in soluble sugar content [39]. In terms of TA, ClO₂(g) treatment conserved a significantly lower concentration of TA throughout the storage (Figure 3E). After 1.5 days, ClO₂(g)-treated mango pulp had 28% and 14% lower TA in contrast with that of untreated and ethephon-treated mangoes (Figure 3E). Moreover, the sugar/acid ratio (TSS/TA) of mangoes treated with ClO₂(g) and ethephon reached 18.89 and 14.95, respectively, on day 2 as compared to 11.91 in the control group. On day 4, the sugar/acid ratio of mangoes in the ClO₂(g) and ethephon groups reached 44.66 and 37.35, respectively, as compared to 30.65 in the control group. 

### 3.3. Sensory Profile and ClO_2_ Residues of Mangoes Fruits

**Figure 4:**
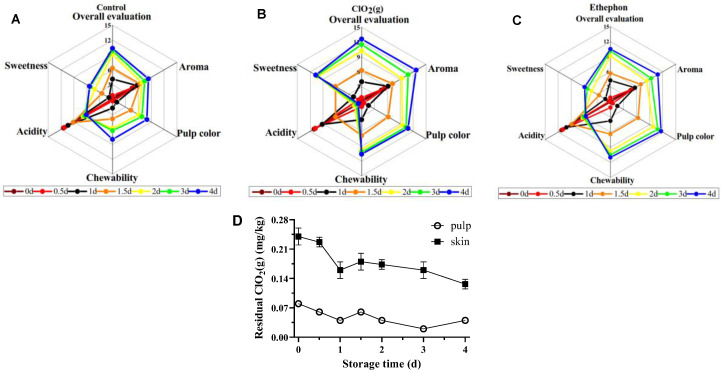
Sensory profiles of untreated (**A**), ClO_2_(g)-treated (**B**), and ethephon-treated (**C**) mangoes during storage at 25 °C. Residual ClO_2_(g) levels in pulp and peel of ClO_2_(g)-treated mangoes during storage at 25 °C (**D**).

The organoleptic characteristic changes of the mangoes during the storage time are shown in Figure 4. The ClO₂(g) samples scored higher in overall appreciation, pulp color, chewiness, and sweetness, whereas they scored lower in sourness compared to the control from day 2 (Figure 4A,B). Notably, mangoes treated with ClO₂(g) had a more prominent sweetness and lower sourness compared to those with ethephon treatment (Figure 4B,C). Collectively, ClO₂(g)-ripened mangoes exhibited the highest sensory attributes compared to those with other treatments.

The residual ClO_2_ levels of the mangoes treated with ClO₂(g) during the storage time are shown in Figure 4D. The residual ClO_2_ content on the skin of mangoes was a maximum of 0.24 mg/kg after ClO₂(g) treatment and gradually decreased to 0.13 mg/kg on the 4th day. For the pulp, the content of ClO_2_ remained in a range of 0.08–0.02 mg/kg during the storage time. The maximum residual disinfectant level goal (MRDLG) in drinking water is 0.8 ppm according to the U.S. EPA [40], which suggests that ClO₂(g) treatment is a safe technique to reduce the time for mangoes to reach the edible window.

### 3.4. Respiration Rate and Ethylene Production

**Figure 5:**
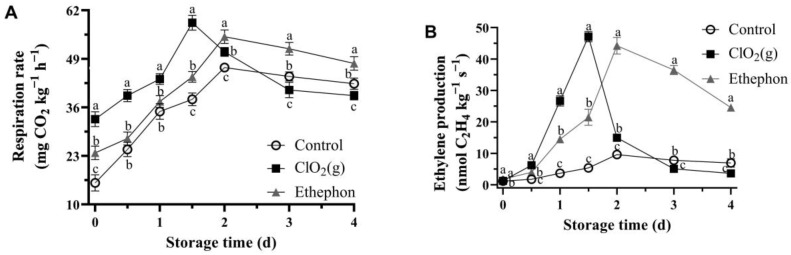
Respiration rate (**A**) and ethylene production (**B**) of untreated, ClO_2_(g)-treated, and ethephon-treated mangoes during storage at 25 °C. Different letters represent statistical differences between groups (*p* < 0.05).

Respiration rate and ethylene production showed a significant increase and then a decrease during the storage time (Figure 5). In general, the respiration rate of mango fruits treated with ClO₂(g) was continuously increased between days 0 and 1.5 and reached a maximum value of 55.68 mg CO_2_ kg^−1^ h^−1^ on day 1.5, followed by a gradual decrease till the end of storage time (Figure 5A). Nevertheless, the respiration rates of mango fruits in the ethephon and control groups were significantly lower than those of the ClO₂(g) group during days 0 and 1.5 and reached the highest values of 54.94 and 46.69 CO_2_ kg^−1^ h^−1^ on day 2, respectively (Figure 5A). The respiration rate of ClO₂(g)-treated mango fruits was significantly lower than that of the ethephon group from day 2 and the control group from day 3 (Figure 5A). 

The ethylene production and respiration rate exhibited a very similar trend. Overall, ethylene production increased significantly with storage time and then progressively decreased till the end of storage time (Figure 5B). The climacteric peak of ethylene culminated on day 1.5 in the ClO₂(g) group (47.12 nmol C_2_H_4_ kg^−1^ s^−1^) and on day 2 in the ethephon (44.25 nmol C_2_H_4_ kg^−1^ s^−1^) and control groups (9.59 nmol C_2_H_4_ kg^−1^ s^−1^) (Figure 5B). Moreover, ethylene production in the ClO₂(g) group was lower than that in the ethephon group on day 2 and lower than that in the control group on day 3. (Figure 5B). These results indicate that ClO₂(g) may promote the production of ethylene, thereby promoting mango ripening.

### 3.5. Transcriptomic Profile of Ripe Mango

**Figure 6:**
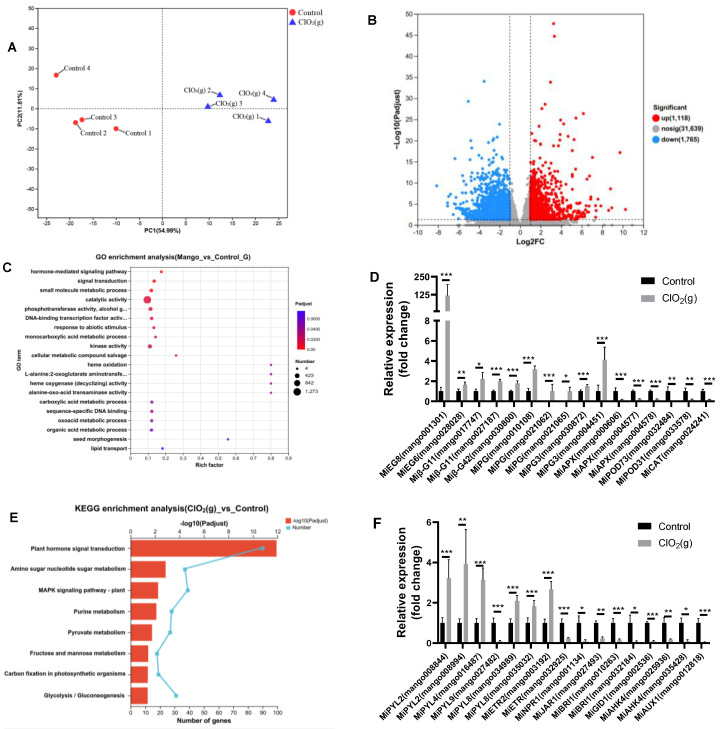
Global gene expression profile of mango pulp treated with and without ClO_2_(g) and stored at 25 °C for 2 days. PCA (**A**) and a volcano plot (**B**) of the transcriptome data; GO enrichment analysis of 2,894 DEGs (**C**); changes of some enzymes related to mango fruit ripening (**D**); KEGG enrichment analysis for 2,894 DEGs (**E**); relative expression of plant hormone receptors (**F**). EG, endoglucanase; β-G, β-glucosidase; PG, polygalacturonase; APX, L-ascorbate peroxidase; POD, peroxidase; CAT, catalase; *n* = 4, * *p* < 0.05, ** *p* < 0.01, *** *p* < 0.001.

According to the physiological and biochemical indices change of Keitt mango, we chose mango samples with 2 days of storage for global transcriptome analysis to unravel the underlying mechanisms of ClO_2_(g) application to accelerate mango fruit ripening. Specifically, high-quality clean data (51.73 Gb) were obtained for eight mango pulp samples, where > 92% of the clean reads were mapped to the mango reference genome (Supplementary Table S2). A total of 25,586 genes were detected in the eight samples. PCA analysis showed obvious differences in overall gene expression between mango pulp treated with and without ClO_2_(g) (Figure 6A). Furthermore, the volcano map showed that 2,894 differentially expressed genes (DEGs) were identified by comparing the group of control and ClO_2_(g) treatment, with 1,123 upregulated and 1,771 downregulated (Figure 6B, Supplementary Table S3). 

GO enrichment analysis showed that the DEGs were mainly functionally associated with catalytic activity (1,273) (Figure 6C, Supplementary Table S4), of which enzymes associated with ripening were significantly regulated in response to ClO_2_(g) treatment. For instance, the genes of enzymes that correlated with fruit softening such as *MiEG8*, *MiEG6*, *Miβ-G11*, *Miβ-G42*, *MiPG*, and *MiPG3* were significantly upregulated after ClO_2_(g) treatment, whereas the enzymes associated with antioxidant damage including *MiAPX*, *MiPOD73*, *MiPOD31*, and *MiCAT* were significantly downregulated (Figure 6D). These were consistent with the previous findings that cellulase and polygalacturonase activities were higher and peroxidase, catalase, and ascorbate peroxidase activities were lower in senescent mangoes [2]. 

**Figure 7:**
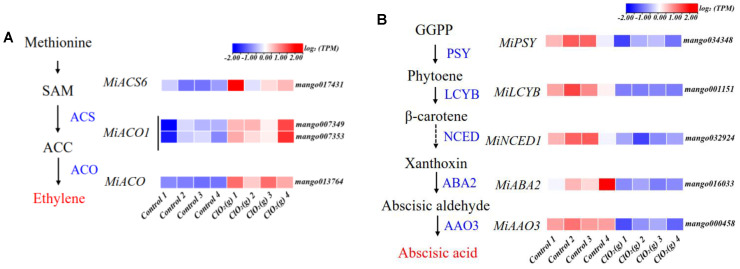

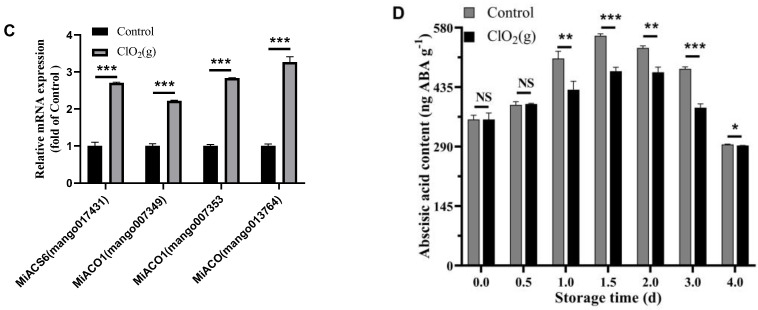
Effect of ClO_2_(g) on the biosynthesis of ethylene and abscisic acid in mango during storage at 25 °C. Ethylene (**A**) and abscisic acid (**B**) biosynthesis pathway and the related gene expression on storage of day 2; (**C**) mRNA expression of *MiACS6*, *MiACO1*, and *MiACO*. (**D**) Abscisic acid content during the storage time. *n* = 4, * *p* < 0.05, ** *p* < 0.01, *** *p* < 0.001; NS, no significance.

KEGG pathway analysis of the DEGs was performed, and the results are shown in Figure 6E. The DEGs were significantly enriched in eight pathways, namely, plant hormone signal transduction, amino sugar and nucleotide sugar metabolism, the mitogen-activated protein kinases (MAPKs) signaling pathway-plant, purine metabolism, pyruvate metabolism, fructose and mannose metabolism, carbon fixation in photosynthetic organisms, and glycolysis/gluconeogenesis (Figure 6E). The plant hormone signal transduction was the pathway most affected by ClO_2_(g) (Figure 6E). Most of the gene expression of ethylene receptors (*MiETRs*) and ABA receptor (*MiPYR/PYL*) were significantly up-regulated after ClO_2_(g) treatment, whereas the receptors for salicylic acid (*MiNPR1*), jasmonic acid (*MiJAR1*), brassinosteroid (*MiBRI1*), gibberellin (*MiGID1*), cytokinin (*MiAHK4*), and auxin (*MiAUX1*) were downregulated (Figure 6F). Genes involved in ethylene biosyntheses such as *miACS6*, *miACO1*, and *miACO* were substantially upregulated with ClO_2_(g) treatment (Figure 7A,B); this was consistent with the increased production of ethylene on day 2 (Figure 5B). Nevertheless, ABA biosynthesis enzymes including *MiPSY*, *MiLCYB*, *MiNCED1*, *MiABA2*, and *MiAAO3* were downregulated (Figure 7C). Moreover, the content of ABA in the mango fruit was lower than in the control group from day 1 to day 4 (Figure 7D). These results suggested that ethylene plays a more important role in mango ripening in response to ClO_2_(g). 

## 4. Discussion

The Keitt mango is a typical climacteric fruit and ripens slowly under natural conditions. This significantly affects the consumer response and therefore the sale of fresh Keitt mangoes. We have recently found that ClO_2_(g) may be a potential technique to reduce the time for Keitt mango to reach an edible window. Keitt mango entered the edible window with a TSS of 14–18% [35,37] and firmness of 25–35 N [35]. In our study, the TSS content of ClO₂(g)-treated mangoes reached 14% on day 1.5, ethephon-treated mangoes on day 2, and the control mangoes on day 3. The firmness of the mangoes treated with ClO₂(g) and ethephon was 32.43 N and 30.28 N on day 2, respectively, while that of the control group was 82.43 N. These changes suggested that ClO₂(g) treatment can accelerate Keitt mango postharvest ripening, as can ethephon treatment. The increase in TSS and the decrease of firmness during ripening were the result of a series of enzyme-mediated softening processes, mainly due to an increase in the activities of β-amylase, galacturonase, and cellulase [41]. Consistently, in our study, fruit-softening genes including *MiEG8*, *MiEG6*, *Miβ-G11*, *Miβ-G42*, *MiPG*, and *MiPG3* were significantly upregulated after ClO_2_(g) treatment. 

The color of Keitt mango pulp is typically an important indicator of ripeness since the color of the peel/skin does not always show a consistent trend during ripening [42]. ClO_2_(g) treatment significantly changed the pulp color of Keitt mango from bright yellow to orange as maturity advanced, which is a color change similar to that of the ethephon treatment. The color change in mango pulp may be due to the accumulation of carotenoids, which are the pigments that make up most of the yellow, orange, and red colors in the fruit [43]. In ClO_2_(g)- and ethephon-treated mango pulp, carotenoid accumulated as maturity advanced, thereby giving the mango pulp a yellower color. Moreover, the increase of carotenoids in ClO_2_(g)-treated mangoes may be due to its response to the oxidative stress of ClO_2_ [44]. 

Sugar/acid ratios are critical components of flavor in ripe mangoes [37]. In the present study, the sugar/acid ratio of mangoes treated with ClO₂(g) (18.89–44.66) was significantly higher than that treated with ethephon (14.93–37.36) and untreated (11.91–30.65) on days 2–4. The sugar/acid ratio of mangoes treated with ClO₂(g) reached 44.66 on day 4, which was close to the sugar/acid ratio of fully ripe Kaew mango of about 50 [45]. Sugar/acid ratios ranging from 23 to 50 were considered high-quality mango fruits [46], which indicated that ClO₂(g) may improve the taste of mango. Consistently, the sensory properties of the ClO₂(g)-treated mangoes were superior to those of ethephon-treated and the control-group mangoes, especially the sweetness was more prominent. The sweetness was attributed to the increase in total soluble sugars and the decrease of TA in the mango treated with ClO₂(g). The increase of total soluble sugar in mango during ripening was mainly due to the biochemical conversion of starch into sugar [47]. In the present study, ClO_2_(g) treatment significantly reduced the starch content of mangoes from day 2 to day 4 compared to the ethephon and control group. Moreover, the relative gene expression of starch-degrading enzymes such as β-amylase was significantly increased in mangoes treated with ClO_2_(g) (Supplementary Table S3). Therefore, ClO_2_(g) treatment mainly enhanced the total soluble sugar content by enzymatically dissolving starch into sugar. TA was mainly ascribed to the accumulation of organic acids in fruit cells [48]. The main organic acids in the Keitt mango were citric acid and malic acid, and the decrease in the TA with ripeness was probably due to their utilization as substrates for gluconeogenesis and respiration [37,49]. ClO_2_(g) treatment reduced the TA during mango ripening compared to the ethephon and control group. Therefore, the reduction of TA content in ClO_2_(g)-treated mangoes may also contribute to the increase in total soluble sugars. 

Fruits commonly suffer weight loss during postharvest ripening [27]. The reduction in fruit weight is mainly related to respiration and moisture evaporation through the peel [50]. Water loss will initiate the wilting of fruit skin and loss in fruit texture, flavor, and saleable weight [51]. In general, most fruits will become unsaleable at a water loss of 5–10% of their initial weight [52]. In our case, ClO_2_(g) and ethephon treatment significantly increased the respiration rate of mangoes as compared to the control group, which led to the weight loss of the fruits. The weight loss of mango fruits treated with ClO_2_(g) and ethephon was similar: both higher than the control group yet lower than 5%. Moreover, the ClO_2_ residues of mango fruits were lower than the maximum residual disinfectant level goal (MRDLG) in drinking water [40]. All the results above suggested that the ClO₂(g) treatment in our study can be a safe technique to reduce the time for mangoes to reach the edible window.

Ethylene is the main hormone that controls the initiation of ripening in climacteric fruits [53]. It functions as a stress hormone under biotic and abiotic stress conditions [54]. Under salinity and other abiotic and biotic stresses, plants tend to produce more ethylene through enhancing ACSs and ACOs [55]. In our case, ClO_2_(g) significantly advanced the onset of the peak of ethylene production by 0.5 days and increased the production of ethylene by 8.79-fold compared to the control group. The genes involved in ethylene biosynthesis including *miACS6*, *miACO1*, and *miACO* as well as the gene expression of ethylene receptors (*MiETR*) were significantly upregulated with ClO_2_(g) treatment. The role of ethylene in fruit ripening has been fully demonstrated [14]. The changes in ethylene accumulation that occur with fruit development greatly affect gene transcription, signaling transduction, and physiological responses and trigger the ripening process [11,12]. Transcription factors in the ethylene pathway, such as ERFs, CNR, and RIN, have been identified to regulate the fruit ripening process by changing attributes including color, aroma, and firmness [56]. Therefore, ClO_2_(g) can regulate fruit ripening by triggering or inducing ethylene biosynthesis. In addition, although ethylene is the main causative agent for ripening in climacteric fruit, recent reports have demonstrated that endogenous ABA can stimulate ethylene production and promote ripening in climacteric fruit such as tomatoes and mangoes [18,57]. In our study, ClO_2_(g) treatment significantly upregulated the gene expression of ABA receptors (*MiPYR/PYL*), which is central to the ABA transduction pathway. ABA binding to the PYR/PYL receptors inactivates PP2C phosphatases, leading to the activation of SnRK2s phosphorylated by the GSK3-like BIN2 kinase, which subsequently leads to the fruit ripening [58]. However, the DEGs associated with ABA biosynthesis as well as the content of ABA were downregulated with ClO_2_(g) treatment. More evidence on the relationship between ABA content and ABA receptor activation is needed. Considering the advanced onset and increased production of ethylene as well as the expression profiles of genes involved in ethylene biosynthesis in mangoes treated with ClO_2_(g), our study supported the results that ethylene may play a major role in regulating the ripening process of mango fruits treated with ClO_2_(g).

## 5. Conclusions

Collectively, our study innovatively utilized ClO₂(g) to prepare ready-to-eat Keitt mango. This is a simple, cost-effective, and safe strategy to reduce the time for Keitt mangoes to reach the edible window. ClO₂(g) modulates Keitt mango postharvest ripening via stimulating endogenous ethylene production, and the sweetness of the mangoes was more prominent compared to the use of exogenous ethephon. Nevertheless, more research is needed to elucidate the underlying mechanism by which ClO₂(g)-treated mangoes are sweeter than those treated with ethephon.

## 6. Future Outlook

Gaseous ClO_2_ is a promising candidate technique in promoting the ripening of mango for sale in the market. The ready-to-eat mango can be an alternative to traditional marketing and have a great potential for fresh consumption and the fresh-cut fruit industry. However, the mechanism of gaseous ClO_2_ to improve the Keitt mango eating quality and how to extend the storage time of ripened mango need further research. This will promote the quality of ready-to-eat mango, reduce postharvest ripening loss, and achieve a high-quality development of the mango industry.

## Data Availability

The data presented in this study are available on request from the corresponding author. The data are not publicly available due to commercial restrictions.

## Supplementary Materials

The following supporting information can be downloaded at: https://www.mdpi.com/article/10.3390/foods13020316/s1, Table S1: Primers used in this study; Table S2: RNA-seq statistics; Table S3: DEGs of ‘Keitt’ fruit among group control vs. ClO₂(g); Table S4: Catalytic activity of DEGs in GO enrichment analysis.
